# Occurrence and Molecular Characteristics of Polerovirus BVG Isolates from Poland

**DOI:** 10.3390/pathogens14111087

**Published:** 2025-10-24

**Authors:** Katarzyna Trzmiel, Aleksandra Zarzyńska-Nowak, Beata Hasiów-Jaroszewska

**Affiliations:** Department of Virology and Bacteriology, Institute of Plant Protection—National Research Institute, Węgorka 20, 60-318 Poznań, Poland; a.zarzynska@iorpib.poznan.pl (A.Z.-N.); b.hasiow@iorpib.poznan.pl (B.H.-J.)

**Keywords:** BVG, barley, wheat, coat protein, genetic variability

## Abstract

Barley virus G (BVG), the species *Polerovirus BVG*, within the genus *Polerovirus* in the family Solemoviridae, represents a new threat to cereal crops in Poland. It was first identified in 2022–2023 using high-throughput sequencing in pooled barley samples exhibiting leaf yellowing and stunting symptoms. The presence of BVG was subsequently confirmed by RT-PCR using diagnostic primers described in the literature. A nucleotide BLAST search of the NCBI database revealed sequence identity ranged from 97.8% to 100%. The final results demonstrated mixed infections involving BVG and luteovirus pashordei, formerly barley yellow dwarf virus—PAS (BYDV-PAS). In 2023–2024, BVG was detected in four additional locations across western, southern, south-eastern, and eastern Poland. The virus was found in co-infection with BYDV-PAS in barley and oat, and with mastrevirus hordei, formerly wheat dwarf virus (WDV) in wheat. Due to the mixed nature of BVG infections, a set of total RNA samples previously isolated from BYDV-infected plants was reanalyzed. RT-PCR results confirmed BVG/BYDV-PAS co-infections in samples collected in 2014–2015, 2018–2019, and 2020–2021. These findings indicate that BVG has been present in Poland for at least 10 years. Molecular characteristics were assessed based on the coat protein gene sequence.

## 1. Introduction

Barley virus G (BVG)*,* the species *Polerovirus BVG*, is classified within the genus *Polerovirus* in the family Solemoviridae. The virus was first isolated and identified in 2016 in South Korea in barley samples exhibiting yellow discoloration of leaf tips and margins [1] and subsequently has also been identified in foxtail millet and proso millet [2,3]. In the following years, BVG was identified in switchgrass in the Netherlands [4], in Kenya [5], in oats, barley and wheat in Australia [6], in various types of millet in Hungary [7], in barley in the United States [8], in maize in Greece [9], in barley in France [10] in wheat in Japan [11], and in weed plants in Germany [12] and in Slovenia [13]. Furthermore, BVG was also identified in a 34-year-old oat herbarium sample in Australia [14]. It has been identified in both symptomatic and asymptomatic plant samples, predominantly in mixed infections with other viruses, exhibiting typical symptoms of yellow dwarf virus infection, which include yellowing or reddening of leaf tips and margins as well as stunting of the entire plant [15]. Mild yellowing symptoms in wheat and barley plants and reddish leaf discoloration in red oat, proso millet, and reddish discoloration of the tassel and corn silk of maize plants, were observed after infection of a BVG infectious clone [16]. However, due to its recent discovery, the economic impact of this virus on crop production across all identified host species and affected regions has not yet been comprehensively evaluated. BVG, like other poleroviruses, is probably not seed-transmissible but is obligatorily transmitted between plants exclusively by aphid vectors in a persistent, non-propagative manner [15]. The two aphid species *Rhopalosiphum padi* and *R. maidis* have been experimentally confirmed as competent vectors. Moreover, even a single viruliferous *R. maidis* aphid is able to initiate an infection [16]. Given the global presence of vectors, BVG is likely to have a wide potential distribution [15]. Although the size of the BVG virion has not yet been experimentally confirmed, it is presumed to be within the 26–30 nm diameter range, consistent with other poleroviruses [17]. The BVG genome contains single-stranded, positive-sense RNA with 5620 nucleotides and six open reading frames (ORFs). ORF0 encodes a viral suppressor of gene silencing; ORF1 encodes a putative polyprotein protein (P1), with conserved serine protease and genome-linked peptide motifs; and ORF2 overlaps with ORF1 and is likely expressed as a fusion protein via a −1 frameshift mechanism to produce the putative RNA dependent RNA polymerase protein. ORF3 encodes the putative capsid protein (CP); ORF4 is embedded inside ORF3 and encodes, in a different reading frame, the putative long-distance movement protein (MP) while ORF5 is expressed from a translational readthrough domain in ORF3 and encodes the ORF3-ORF5 readthrough protein required for aphid transmission [1,15].

To date, most studies concerning BVG have focused solely on the detection and analysis of field samples. Ericson et al. [16] investigate the biological properties of the virus. Therefore, the knowledge regarding BVG diversity and phylogenetic relationships among isolates is rather limited [18].

Cereals constitute the main group of cultivated plants in Poland. The total area of major cereal crops exceeds 7 million hectares (https://stat.gov.pl/en/topics/statistical-yearbooks/statistical-yearbooks/statistical-yearbook-of-agriculture-2023,6,18.html, accessed on 10 May 2024). In light of recent detections of BVG infections in Europe [10] and the possible presence of the virus in cereal crops in Poland, preliminary studies have been undertaken. In this study, we described the detection of BVG isolates across various regions of the country, in samples collected during cereal surveys conducted in two consecutive growing seasons, 2022–2023 and 2023–2024, as well as from collected archival samples dating back to 2014–2015. Comprehensive analysis of BVG population diversity based on coat protein sequences, recombination events, and phylogenetic relationship analysis has also been carried out. The results of this study expand the knowledge regarding BVG’s occurrence, distribution, and genetic variability.

## 2. Materials and Methods

### 2.1. Materials

Plant samples of the main cereal species (wheat, barley, rye, triticale, and oat) with stunting and leaf discoloration symptoms were collected from experimental fields, as well as crop production areas located in different regions of Poland. In 2022–2023, season 15, plant samples were gathered including winter barley (9), winter wheat (3), and triticale (2). Analogically, in 2023–2024, in total, 51 plant samples were collected: winter wheat (30), winter barley (18), rye (2), and oat (1). The plant samples were stored at −80 °C until further analysis. Moreover, due to the mixed nature of BVG infections, a set of total RNA samples previously isolated from BYDV-infected plants was also reanalyzed. For this reason, 10, 8, and 15 samples originating from the periods 2014–2015, 2018–2019, and 2020–2021, respectively, were used. Additionally, insect samples (*R. maidis* and *Sitobion avena* )collected in 2023–2024 from BVG-infected barley plants growing in the Silesia region were used in this study. The insects were preserved in 70% ethanol until they were used.

### 2.2. RNA Isolation

The total RNA extractions from plants (100 mg plant tissue per sample) were performed using TRI Reagent™ (Thermo Fisher Scientific, Waltham, MA, USA). For nucleic acid extractions from insect samples (5 individuals per sample), the Total RNA Purification Kit (Novazym, Poznań, Poland) was used. The procedures were conducted in accordance with the manufacturer’s protocols. The concentration and quality of obtained RNAs were estimated spectrophotometrically by an ND 2000 spectrophotometer (Nanodrop Technologies, Thermo Fisher Scientific, Waltham, MA, USA) and fluorometrically with a Qubit 3 fluorometer (Thermo Fisher Scientific). The integrity and length of extracted RNA samples were analyzed by electrophoresis in 1% agarose gel with Midori Green DNA Stain (NIPPON genetics Europe GmbH, Düren, Germany) and viewed under UV light. For HTS analysis, RNA was isolated from one pooled sample (leaves from field barley collected in 2022–2023), while for screening tests, RNA was obtained from individual plant and aphid samples (from the periods 2022–2023 and 2023–2024, as well as samples previously collected in 2014–2015, 2018–2019, and 2020–2021).

### 2.3. High-Throughput Sequencing (HTS)

The HTS reaction was performed according to the procedure described earlier [19]. In the first step, the ribosomal RNA from the analyzed RNA sample was removed using the Ribo-off rRNA depletion kit (Vazyme, Nanjing, China). Next, the sequencing library was prepared with the VAHTS^®^ Universal V6 RNA-seq Library Prep kit for Illumina (Vazyme). HTS was carried out using the NovaSeq 6000 platform with 150-nucleotide (nt) paired-end chemistry by weSEQ.IT company (Rybnik, Poland,). Obtained reads were trimmed with Skewer (version 0.2.2) and fastp software (version 0.21.0). Afterwards, bioinformatic analyses, generation of consensus sequences, and mapping were performed using the CLC Genomics Workbench (version 20.0.3) (Qiagen, Hilden, Germany).

### 2.4. Reverse Transcriptase Polymerase Chain Reaction (RT-PCR)

The presence of BVG identified by the Illumina platform was confirmed using RT-PCR and primer pairs BVG-F/BVG-R amplifying a 394 bp product, published by Svanella-Dumas et al. [10]. For cDNA synthesis, SuperScript IV and random hexamers (Thermo Fisher Scientific) were used, with the original RNA extracts from individual barley samples. The cDNA was used as a template for PCR using the Dream Taq Green PCR Master Mix 2X (Thermo Fisher Scientific). The reactions were conducted according to the manufacturer’s instructions.

For screening tests, newly designed primer pairs BVGcp2-F/BVGcp2-R (5′-CTTGCAGCCATAGATTGGA-3′/5′-TGATTGAATTCGGCTTTCC-3′), which amplify an 850 bp PCR product containing the complete CP gene sequence, were used. The oligonucleotide sequences were designed with Primer3 software (version 4.1.0) (https://primer3.ut.ee//, accessed on 12 September 2023) [20] based on the reference sequence of BVG-Gimje (KT962089). RT-PCR reactions were performed in a total volume of 10 µL using the RevertAid Reverse Transcriptase (200 u/µL) and the Dream Taq Master Mix 2X (Thermo Fisher Scientific), according to the manufacturer’s instructions. The thermal conditions of the reactions were as follows: reverse transcription at 42 °C for 20 min, followed by initial denaturation at 94 °C for 3 min then 40 cycles of denaturation at 94 °C for 30 s, annealing at 50 °C for 30 s, elongation at 72 °C for 60 s, and a final elongation at 72 °C for 7 min. The presence of specific RT-PCR products was determined by electrophoresis on 1% TBE agarose gel stained with Midori Green DNA stain (Nippon Genetics Europe Gmbh) and visualized under UV light.

Additionally, infections caused by a group of barley yellow dwarf viruses (BYDVs), which represent the main viral threat to cereal crops in Poland [21,22], were confirmed using one-step RT-PCR with BYcp-F/BYcp-R primers [23] followed by a restriction fragment length polymorphism (RFLP) analysis. This assay, developed for BYDVs typing, allows the detection and discrimination of three species: luteovirus pavhordei (formerly barley yellow dwarf virus-PAV, BYDV-PAV), luteovirus mavhordei (formerly barley yellow dwarf virus-MAV, BYDV-MAV), and luteovirus pashordei (formerly barley yellow dwarf virus-PAS, BYDV-PAS) [24].

The detection of viruses in aphids was carried out according to the procedure described by Strażyński et al. [25]. The primers Act1/Act2 [26], which amplify a 320 bp fragment of a conserved region of insect actin, were used as an internal control for virus detection in aphids.

### 2.5. Duplex Immunocapture–Polymerase Chain Reactions (Duplex-IC-PCR)

In order to determine the co-infection of BVG with mastrevirus hordei (formerly wheat dwarf virus, WDV), Duplex-IC-PCR reactions using the primer pairs WDV-H-F/WDV-H-R and WDV-T-F/WDV-T-R were carried out on plant sap samples, as previously described [27]. The Duplex-IC-PCR results were confirmed by Sanger sequencing.

### 2.6. DNA Sequencing

The specific RT-PCR and Duplex-IC-PCR products of the appropriate sizes were excised from agarose gels, purified with Wizard^®^ SV Gel and PCR Clean-Up System (Promega, Madison, WI, USA), and then used for direct Sanger sequencing with specific primer pairs (mentioned above) by Genomed S.A. (Warsaw, Poland). The nucleotide sequences were initially assembled using the BioEdit software, version 7.7.1 [28], and the resulting consensuses of sequences were subsequently verified through a Standard Nucleotide BLAST search (BLAST, http://blast.ncbi.nlm.nih.gov/Blast.cgi, accessed on 15 May 2024). The obtained nucleotide sequences of the CP gene were deposited in the GenBank database of the National Center for Biotechnology Information (NCBI) under the accession numbers listed in Table 1.

### 2.7. Genetic Variability Assessment and Phylogenetic Analysis

The bioinformatics analyses were performed based on the coding sequence of the CP gene of BVG. In this study, the nucleotide sequences of 14 newly obtained Polish BVG isolates (Table 1), as well as 30 additional sequences retrieved from the GenBank database (Table S1), were used. Among them, 12 CP gene BVG sequences belong to the European group (from France, Germany, Greece, Hungary, the Netherlands, and Slovenia), 5 originated from Australia, 1 originated from Japan, 1 originated from the USA, and the remaining 11 sequences originated from South Korea (Table S1). The multiple sequence alignments were conducted using the BioEdit Sequence Alignment Editor with ClustalW [28]. Sequence identity matrices were displayed using BioEdit and Sequence Demarcation Tool Version 1.2 (SDTv1.2) [29]. Before the phylogenetic analyses, the occurrence of potential recombination events among the studied BVG isolates was assessed using the RDP, GENECONV, Chimera, MaxChi, BootScan, SiScan, 3Seq, and LARD methods implemented in Recombination Detection Program version 4 (RDP4), with default settings [30]. Recombination events were considered significant if at least four of the aforementioned detected them with *p*-value below 0.05 and if there was additional phylogenetic evidence supporting recombination. Phylogenetic analyses were performed on the amplified 594-nt coding sequence of the BVG CP gene using the Maximum Likelihood method in MEGAX [31]. The Kimura 2-parameter model and a gamma distribution (K2+G) were applied for nucleotide sequences, and the Jones–Taylor–Thornton (JTT) model was used for deduced amino acids (aa) sequences. Branch support was assessed with 1000 bootstrap replicates.

## 3. Result

### 3.1. Virus Identification by Illumina Sequencing

The analysis of a pooled barley plant sample used for HTS indicated the presence of different virus species. The mixed infections included BYDV-PAV, BYDV-PAS, WDV, and tritimovirus tritici (formerly wheat streak mosaic virus, WSMV), which had already been reported in cereal crops in Poland [21,27,32], as well as BVG, which had not been previously detected in the country. The raw sequencing data was deposited in the Sequencing Read Archive (SRA) under accession number SRR35566536 within BioProject PRJNA1232637. A detailed description of HTS results is presented in Table 2.

### 3.2. BVG Occurrence Assessment

**Figure 1:**
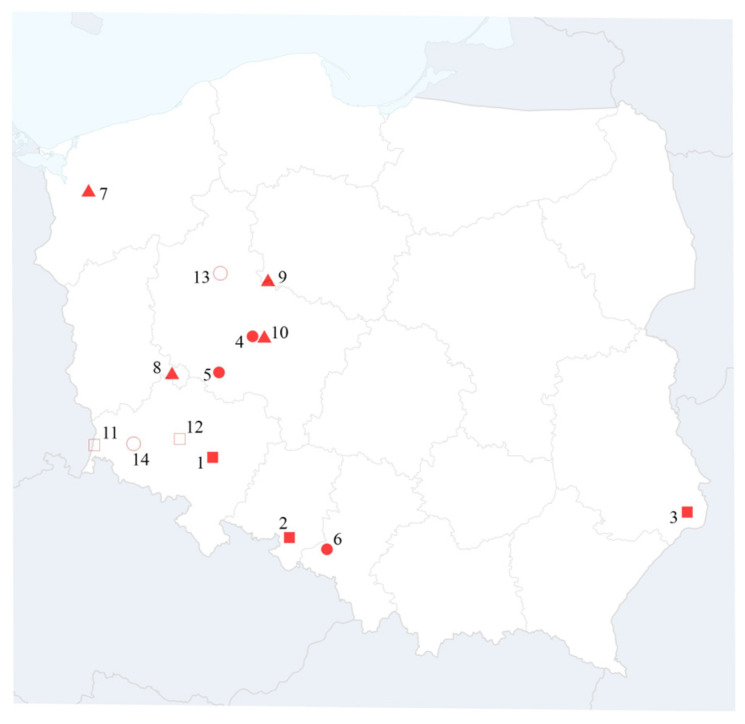
Locations of winter barley plants infected with BVG: 1—BVG-ZYB; 2—BVG-POH; 3—BVG-LP1; 4—BVG-WG1; 5—BVG-CHW; 6—BVG-Sosn; 7—BVG-H; 8—BVG-VH3; 9—BVG-Kow; 10—BVG-WG2; 11—BVG-Koz; 12—BVG-P3J; 13—BVG-Orlowo; 14—BVG-Zlotoryja. The Polish isolates are marked as follows: BVG collected in 2023–2024 with a red square, 2022–2023 with a red circle, 2020–2021 with a red triangle, 2018–2019 with a white square, and 2014–2015 with a white circle.

**Figure 2:**
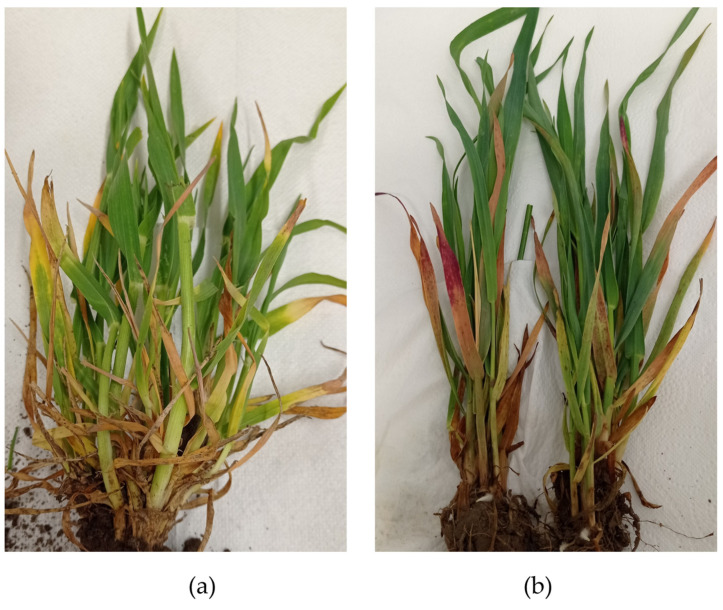
Symptoms of co-infection with BYDV-PAS and BVG observed in winter barley (**a**) and oat (**b**) plants.

The results of this study confirmed the presence of BVG in 3 out of 9 tested barley plants collected in 2022–2023, as well as in 2 out of 30 tested wheat plants, 3 out of 18 barley plants, and 1 oat plant sampled in 2023–2024. Furthermore, reanalysis of RNA samples extracted from plants infected with BYDV-PAS revealed the presence of BVG in 2 out of 10 samples from the period 2014–2015, 2 out of 8 samples from the period 2018–2019, and 4 out of 15 samples from the period 2020–2021. Fourteen isolates, each with the complete coding sequence of the CP gene, were selected for further analyses. They were collected from 14 different locations, mostly in western Poland (Figure 1), from plants exhibiting symptoms characteristic of BYDV infection (Figure 2).

In all analyzed cases, BVG was detected in mixed infections with other viruses, primarily with BYDV-PAS (12 samples) and WDV (2 samples) (Table 1).

Moreover, BVG was also isolated from two aphid species (*R. maidis* and *S. avenae*) collected in 2023–2024 from barley plants originating from southwestern Poland.

### 3.3. Genetic Variability of the BVG Population

**Figure 3:**
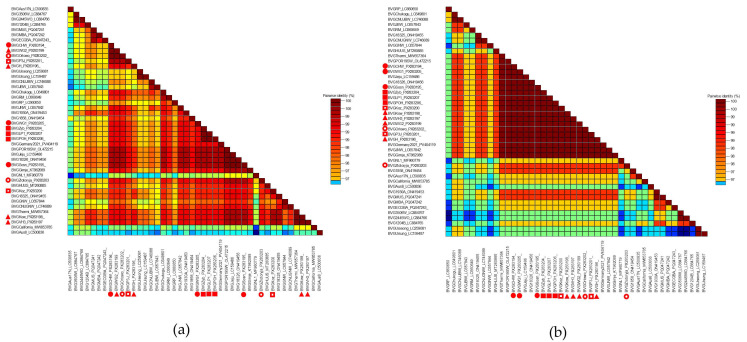
Two-dimensional, color-coded identity matrix visualizations of nucleotide (**a**) and amino acid (**b**) sequences of 44 BVG isolates, generated for the CP gene. The matrices were created using SDT v.1.2 with default program settings. Each cell represents the percentage identity between two sequences, as indicated by the color scale on the right side of each matrix. Polish isolates are marked as follows: BVG collected in 2023–2024—red square, 2022–2023—red circle, 2020–2021—red triangle, 2018–2019—white square, and 2014–2015—white circle.

Genetic variability within the BVG population was assessed based on the coding sequence of the CP gene. The 14 Polish isolates (Table 1) originating from different regions of the country and hosts were closely related to each other, sharing 99.3% to 100% and 99.4% to 100% nucleotide and amino acid identity, respectively. An analogous comparison of the studied BVG isolates with those from the GenBank database (Table S1) revealed a variable level of similarity. The lowest nucleotide sequence similarity (97.9%) was observed for the Australian isolate BVG-Aus8 (LC500836). In contrast, 100% identity was observed between the isolates BVG-Kow (PX283198) and BVG-VH3 (PX283197) and the Greek isolate BVG-Thermi (MW657364), as well as between the isolate BVG-Sosn (PX283195) and the isolates BVG-POR19SW (OL472215) from Slovenia, BVG-Jeju (LC159486) from South Korea, and BVG-18-326 (ON4149456) from France. Subsequent comparative analyses based on amino acid sequences revealed the lowest similarity (97.9%) between BVG-Zlotoryja (PX283203) and the Australian isolates BVG-3-506W and BVG-2-445WO (LC884767, LC884766). In contrast, 100% identity was observed between the analyzed group of Polish isolates (excluding BVG-Zlotoryja) and the isolates BVG-Gimje (KT962089), BVG-JNW (LC657842), and BVG-Jeju (LC159486) from South Korea; BVG-Thermi (MW657364) from Greece; BVG-POR19SW (OL472215) from Slovenia; BVG-18-326 (ON4149456) from France; and BVG-Germany-2021 (PV404119). The visualization of nt and aa sequence identity for all analyzed BVG isolates, performed by SDTv1.2, is shown in Figure 3a,b. Detailed numerical data are provided in the Supplementary Materials (Table S2).

### 3.4. Phylogenetic Analysis

**Figure 4:**
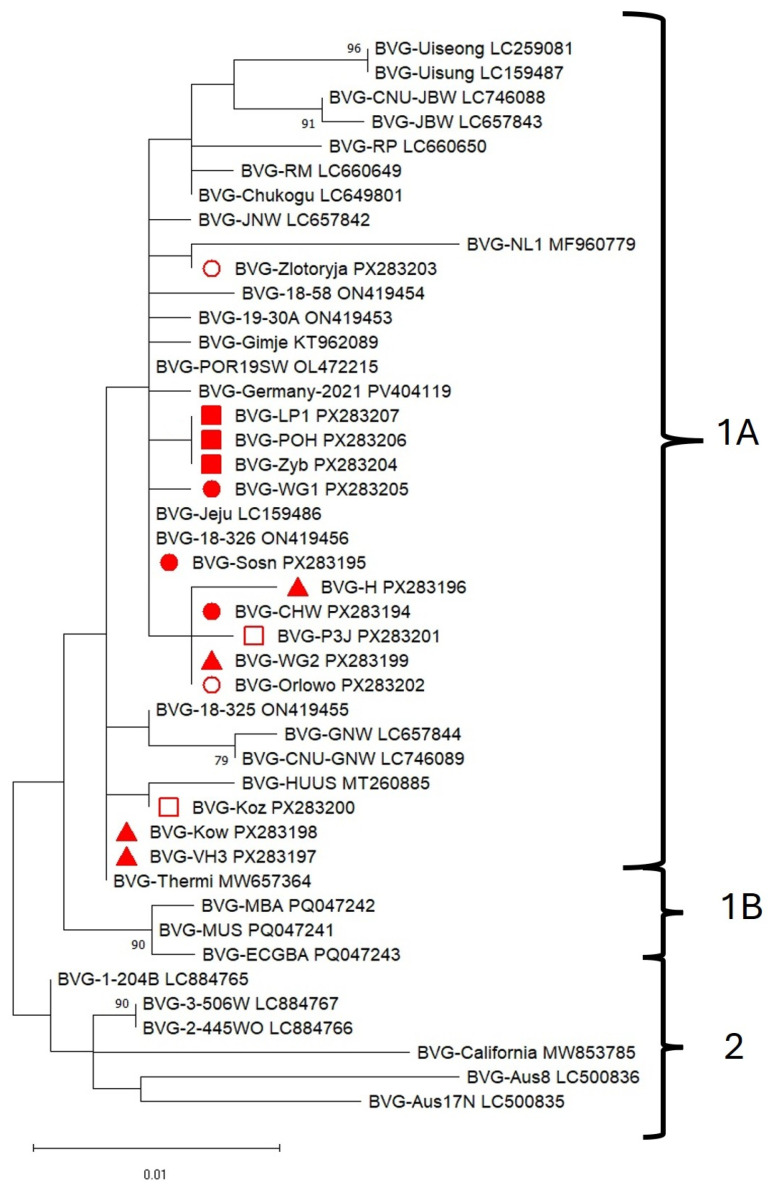
Maximum likelihood phylogenetic tree of 44 polerovirus BVG isolates, constructed based on the nucleotide sequence of the CP gene. The names and GenBank accession numbers of the isolates are provided. Bootstrap values (from 1000 replicates) are indicated at major nodes, shown only when greater than 70%. The Polish isolates are marked as follows: BVG collected in 2023–2024 with a red square, 2022–2023 with a red circle, 2020–2021 with a red triangle, 2018–2019 with a white square, and 2014–2015 with a white circle.

No recombination events were detected in the analyzed sequence set using RDP4 software; consequently, 44 isolates (Table 1 and Table S1) were included in the phylogenetic analysis. The phylogenetic analysis of ORF3, which encodes the CP gene, is presented in Figure 4 and Figure S1. The BVG topology was composed of two main clades, based on nucleotide sequences (Figure 4).

The largest clade, 1A, consisted of a group of European isolates along with those from South Korea and Japan. Clade 1B included three Hungarian isolates collected in 2021, while clade 2 was formed by virus isolates from the USA and Australia. All BVG isolates obtained in this study clustered within clade 1A; however, they were distributed across different branches. BVG isolates collected in 2023–2024 (BVG-LP1, BVG-POH, and BVG-Zyb) clustered together, whereas those originated in the period 2022–2023 (BVG-WG1, BVG-CHW and BVG-Sosn) were positioned separately. Two Polish isolates from the period 2020–2021 (BVG-Kow and BVG-VH3) grouped with the BVG-Thermi isolate from Greece, while the remaining two (BVG-H and BVG-WG2) clustered with other Polish isolates identified in 2022–2023 (BVG-Sosn), 2018–2019 (BVG-P3J), and 2014–2015 (BVG-Orlowo). BVG-Koz (2018–2019) grouped with BVG-HUUS from Hungary, whereas BVG-Zlotoryja (2014–2015) clustered with BVG-NL1 from the Netherlands. Phylogenetic analyses based on amino acid sequences (Figure S1) revealed a different topological arrangement compared to analyses based on nucleotide sequences.

Polish isolates clustered within the same branch, except BVG-Zlotoryja, which grouped with BVG-NL1. Together, they formed clade 1A. Clade 1B comprised isolates originating from Australia, France, Hungary, and South Korea. BVG-California from the USA formed clade 1C with isolates from South Korea and Japan. Clade 2 included isolates from Australia and Hungary, in addition to one French isolate.

## 4. Discussion

HTS techniques can overcome the limitations of classical serological and molecular diagnostic methods. It is a powerful technology for accurately identifying viral entities in tested plants without any prior knowledge, providing a comprehensive overview of the plant’s viral phytosanitary status [33,34,35]. Recently, different sequencing platforms have been successfully employed for the detection of viruses in cereal crops. Many of these studies identified well-known viral pathogens, such as yellow dwarf virus complex [36,37,38], bromovirus BMV (formerly brome mosaic virus, BMV) [39], and WSMV [40]; however, the detection and identification of novel threats for cereal crops have also become increasingly important. One of them is BVG, which has been detected in mixed infection with other cereal viruses so far [1,6,10,18]. The results of our study using HTS confirmed the presence of four virus species previously reported in Poland, BYDV-PAV, BYDV-PAS, WDV, and WSMV [21,27,32], as well as the occurrence of a new virus—BVG.

BVG was first identified in 2016 in South Korea in barley samples exhibiting symptoms similar to those of barley yellow dwarf disease. Up to date, it was detected in cultivated cereal crops and various pooled weed species [2,3,4,7] that serve as a reservoir for the virus in the environment. The screening results found in our study demonstrated the presence of BVG in barley and wheat plants as well as in aphid samples (*R. maidis* and *S. avenae*) collected from barley plants in which BVG was detected (Table 1). These results for *R. maidis* are unsurprising, as this species has been previously confirmed as an efficient BVG vector [16]. Although *S. avenae* is listed among the many vectors of the YDV complex [41], confirmation of BVG transmission by this species requires additional experiments. In our paper, we described the detection of the virus in newly (2022–2023, 2023–2024) collected barley and wheat samples as well as in archived barley samples from the years 2014–2015, 2018–2019, and 2020–2021. This finding indicates that BVG remained undetected in the Polish crops for at least 10 years. Similarly, in Australia, BVG was detected in a 34-year-old oat herbarium sample [14], which suggested that the virus had been present in monocotyledonous plants worldwide much earlier than originally assumed. Moreover, its true origin remains unclear.

In most of the above-mentioned cases, BVG was present in mixed infections. Such situations result from the limitations of commonly used classical serological and molecular diagnostic techniques in detecting multifactorial infections and highlight the need to complement them with modern approaches such as HTS. Despite numerous recent studies utilizing HTS technology that confirm the presence of BVG in new locations, information on the global diversity of the BVG population remains limited. As previously shown, the CP is the main component of the plant virus virion, which is involved in nearly all stages of the viral infection cycle; therefore, it remains the focus of considerable research interest [42]. In the present study, 14 new coding sequences of the CP gene were obtained and compared with 30 sequences previously deposited in the GenBank database. The results indicated a high level of identity for the nucleotide and protein sequences from 96.9% to 100% and from 97.4% to 100%, respectively (Table S2). The comparative analysis revealed the lowest similarity values with the oldest known BVG isolate, BVG-Aus8, collected in Australia in 1985. Apart from this case, no significant influence of origin (location, host, or collection date) was observed on the level of genetic diversity within the BVG CP region. This suggests a relatively stable genetic structure of the virus across different environments and hosts. Indeed, the complete nucleotide sequence identity (100%) among isolates from different countries, hosts, and years indicates that the BVG CP gene is highly conserved. Nevertheless, the absence of clear geographical or temporal patterns should be interpreted with caution, as the available dataset is limited in both size and representativeness. The phylogenetic analysis based on the coding sequence of the BVG CP gene showed that the group of the Polish BVG isolates is more similar to those from Europe (Germany, the Netherlands, France, Greece, Slovenia, and Hungary) and Asia (South Korea, and Japan) than to the isolates from the USA and Australia. The same tendency and the presence of two main clades were reported by Lee et al. [18]. The inclusion of a greater number of available sequences led to the emergence of internal subdivisions and enabled the identification of two subgroups (1A and 1B) based on nucleotide sequences and even three subgroups (1A, 1B, and 1C) based on amino acid sequence analysis. All isolates from Poland clustered within clade 1A. However, differences in the coding sequence of the CP gene and the phylogenetic relationships of the analyzed group of Polish BVG isolates suggest the possibility of two independent introductions of BVG into the country. This assumption may be confirmed by further studies based on longer fragments or full-length viral genomes.

The presented study is the first report to provide the identification and molecular characterization of the Polish BVG isolates, as well as an updated description of the phylogenetic relationships within the global BVG population. Based on the obtained results, it can be assumed that this species most frequently occurred in co-infection with viruses associated with barley yellow dwarf disease.

## Figures and Tables

**Table 1:** Description of the Polish polerovirus BVG isolates used in this study.

Name of Isolates	Host	Co-Infection	Geographical Origin	Date of Collection	GenBank Accession Number
BVG-WG1	barley	BVG/BYDV-PAS/BYDV-PAV/WDV	Poland: Greater Poland	2023	PX283205
BVG-CHW	barley	BVG/BYDV-PAS	Poland: Greater Poland	2023	PX283194
BVG-Sosn	barley	BVG/BYDV-PAS	Poland: Silesia	2023	PX283195
BVG-Zyb	barley	BVG/BYDV-PAS	Poland: Lower Silesia	2024	PX283204
BVG-POH	wheat	BVG/BYDV-PAS	Poland: Opole	2024	PX283206
BVG-LP1	wheat	BVG/WDV	Poland: Lublin	2024	PX283207
BVG-H	barley	BVG/BYDV-PAS	Poland: Western Pomerania	2021	PX283196
BVG-VH3	barley	BVG/BYDV-PAS	Poland: Kujavia and Pomerania	2021	PX283197
BVG-Kow	barley	BVG/BYDV-PAS	Poland: Lubusz	2021	PX283198
BVG-WG2	barley	BVG/BYDV-PAS	Poland: Greater Poland	2021	PX283199
BVG-Koz	barley	BVG/BYDV-PAS	Poland: Lower Silesia	2019	PX283200
BVG-P3J	wheat	BVG/BYDV-PAS	Poland: Lower Silesia	2019	PX283201
BVG-Orlowo	barley	BVG/BYDV-PAS	Poland: Greater Poland	2015	PX283202
BVG-Zlotoryja	barley	BVG/BYDV-PAS	Poland: Lower Silesia	2015	PX283203
BVG-S-RM	corn leaf aphid	BVG/BYDV-PAS	Poland: Silesia	2023	PX378101
BVG-S-SA	English grain aphid	BVG/BYDV-PAS	Poland: Silesia	2023	PX378102

**Table 2:** BVG identified using high-throughput sequencing (HTS) with corresponding original host plant species, test plant species, total number of raw reads, percent of reference genome covered by reads, the number of reads mapped for the individual virus reference sequences, and average depth of coverage.

Host Plant	Number of Total Raw Reads	Percent of Reference Genome Covered by Reads	Number of Reads Mapped to Corresponding Reference Sequence from Viral RefSeq	Average Depth of Coverage for Corresponding Viral Species	Name of Identified Virus
barley	48,743,038	87%	4890	235.00	BVG
barley	48,743,038	95.7%	1445	22.72	WSMV
barley	48,743,038	96.7%	360,188	9030.39	BYDV-PAV
barley	48,743,038	94.3%	822,505	21,152.19	BYDV-PAS
barley	48,743,038	99.9%	66,402	3530.23	WDV

## Data Availability

The datasets generated during the current study are available from the corresponding author upon reasonable request.

## Supplementary Materials

The following supporting information can be downloaded at https://www.mdpi.com/article/10.3390/pathogens14111087/s1, Table S1. Description of the polerovirus BVG isolates from GenBank used in this study, Table S2. Sequence identity matrix for nucleotide of amino acid sequence of the BVG CP gene, Figure S1. Maximum likelihood phylogenetic tree of 44 polerovirus BVG isolates, constructed based on the deduced amino acid sequence of the CP. The names and GenBank accession numbers of the isolates are provided. Bootstrap values (from 1000 replicates) are indicated at major nodes, shown only when greater than 70%. The Polish isolates are marked as follows: BVG collected in 2023–2024 with a red square, 2022–2023 with a red circle, 2020–2021 with a red triangle, 2018–2019 with a white square, and 2014–2015 with a white circle.
