# Supplementary material for: Occurrence and Molecular Characteristics of Polerovirus BVG Isolates from Poland
Source: Pathogens. 2025 Oct 24;14(11):1087. doi: 10.3390/pathogens14111087 (PMC12655003; doi:10.3390/pathogens14111087)
Supplement: Supplementary file 1 [file pathogens-14-01087-s001.zip › Table S1.pdf]

Table S1. Description of the polerovirus BVG isolates from GenBank used in this study

Name of isolates	Host	Geographical origin	Date of collection	GenBank accession number
BVG-Gimje	barley	South Korea	2015	KT962089
BVG-Uisung	proso millet	South Korea	2015	LC159487
BVG-Uiseong	proso millet	South Korea	2015	LC259081
BVG-Jeju	foxtail millet	South Korea	2015	LC159486
BVG-JBW	wheat	South Korea	-	LC657843
BVG-GNW	wheat	South Korea	-	LC657844
BVG-CNU-GNW	wheat	South Korea	2021	LC746089
BVG-JNW	wheat	South Korea	2022	LC657842
BVG-CNU-JBW	wheat	South Korea	2021	LC746088
BVG-RM	corn leaf aphid	South Korea	-	LC660649
BVG-RP	bird cherry-oat aphid	South Korea	-	LC660650
BVG-NL1	switchgrass	Netherlands	2016	MF960779
BVG-18-325	barley	France: Cher	2018	ON419455
BVG-18-58	barley	France: Tarn	2018	ON419454
BVG-18-326	barley	France: Cher	2018	ON419456
BVG-19-30A	barley	France: Rhone	2019	ON419453
BVG-HUUS	wheat	Hungary: Keszthely-US	2019	MT260885
BVG-MUS	proso millet	Hungary: Keszthely	2021	PQ047241
BVG-MBA	proso millet	Hungary: Keszthely	2021	PQ047242
BVG-ECGBA	barnyard grass	Hungary: Keszthely	2021	PQ047243
BVG-Thermi	maize	Greece	2019	MW657364
BVG-California	barley	USA	2018	MW853785
BVG-Aus8	barley	Australia	1985	LC500836
BVG-Aus17N	oat	Australia	2016	LC500835
BVG-3-506W	wheat	Australia: Victoria	2020	LC884767

BVG-1-204B	barley	Australia: Victoria	2020	LC884765
BVG-2-445WO	wild oat	Australia: Victoria	2020	LC884766
BVG-POR19SW	pooled weed plants	Slovenia	2019	OL472215
BVG-Germany-2021	pooled weed plants	Germany: Hallertau	2021	PV404119
BVG-Chugoku	wheat	Japan: Chugoku	2019	LC649801
