# Supplementary figures and images for: Occurrence and Molecular Characteristics of Polerovirus BVG Isolates from Poland
Source: Pathogens. 2025 Oct 24;14(11):1087. doi: 10.3390/pathogens14111087 (PMC12655003; doi:10.3390/pathogens14111087)

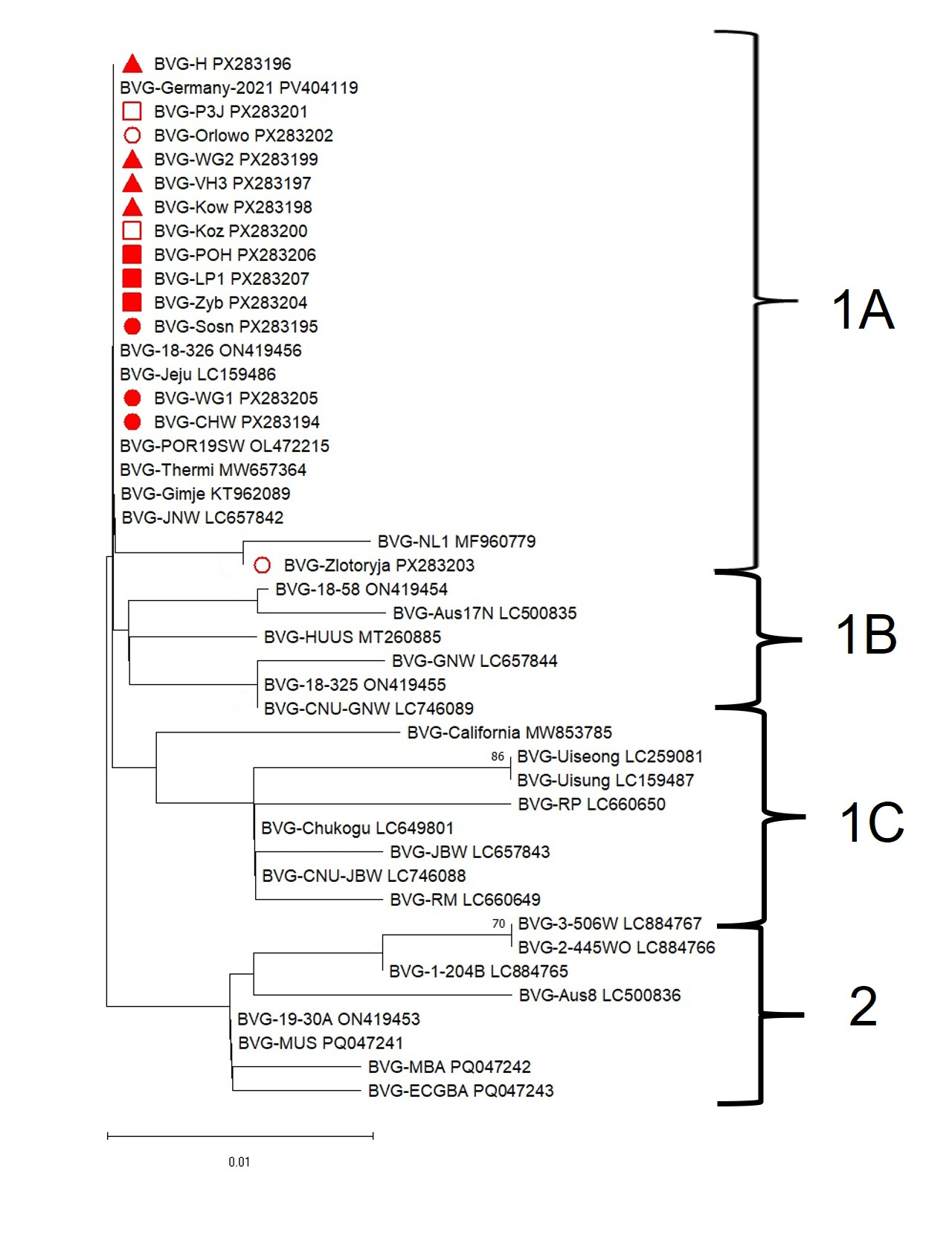

Supplement: Supplementary file 1 [file pathogens-14-01087-s001.zip › Figure S1.tif]
